# The contribution of family physicians to residential mental health care during the COVID-19 pandemic in Tshwane District, South Africa

**DOI:** 10.4102/phcfm.v13i1.3045

**Published:** 2021-07-23

**Authors:** Annelet Kruger, Owen O. Eales, Sanet Jansen van Vuuren

**Affiliations:** 1 Department of Family Medicine, Faculty of Health Sciences, University of Pretoria, Pretoria, South Africa; 2 Kungwini Welfare Organisation, Zwavelpoort, Pretoria, South Africa

**Keywords:** family medicine, coordination of care, virtual, health catchment area, WhatsApp, Zoom

## Abstract

During the start of the first wave of the coronavirus disease 2019 (COVID-19) pandemic, two family physicians in Tshwane, South Africa, reviewed the people at high-risk within their Health Catchment Area. The largest residential mental health care facility in Gauteng fell under their care, and they were responsible for providing care and support to this facility. Family physicians have to lead the primary care team and simultaneously take care of the well-being of their team members. This report discusses how these family physicians used digital platforms and virtual care to successfully coordinate and manage the response to an outbreak of COVID-19 at this mental healthcare facility.

## Introduction

The concept of Health Catchment Areas (HCA), as a defined geographic portion of the Health District, has been used by the Department of Family Medicine at the University of Pretoria (UP) to divide Tshwane District into manageable areas governed by family physicians in primary care. The family physician takes responsibility for all primary care facilities, old age homes, special needs facilities and health related activities in this area. The family physician previously fulfilled all their different roles through being physically present and moving around the HCA. As a result of the coronavirus disease 2019 (COVID-19) pandemic, the key roles of the Family Physician (clinician, consultant, capacity-builder, clinical trainer, leader of clinical governance, and champion of community orientated primary care [COPC]) had to be applied in a new context.^1^

## Background

This report describes the virtual support provided by two family physicians to Paul Jungnickel Home (PJH), a mental healthcare institution in their HCA in Tshwane, during the first wave of the pandemic in 2020. Paul Jungnickel Home is a residential care facility that offers medical and residential care to 160 severe and profoundly disabled adults. The Department of Family Medicine at UP has given clinical support and coordinated care for the home since 2010 and provided on-site support during two Shigella outbreaks. The COVID-19 pandemic presented the healthcare team at PJH with some very specific challenges. Patients with severe intellectual impairments struggled to adhere to general COVID-19 prevention measures such as wearing mask, social distancing and using hand sanitizer. Many of these patients had comorbidities, limited mobility and obesity. Because of these factors, the team expected rapid spread of the virus and a high mortality rate. In addition, these patients would not be prioritised for intensive care or ventilation, particularly in this resource limited setting.

An on-site facility readiness assessment and disaster management plan were completed in April 2020, after the facility went into a strict lockdown. Everyone realised that it would not be business as usual, and that technology would play a big role in providing support.

## Virtual coordination of care

Telemedicine and the use of digital health technologies have long been discussed as a potential solution to many challenges, including primary care. The COVID-19 pandemic dramatically catalysed the uptake of these technologies in a matter of weeks.^2^ Telemedicine assisted in providing early interventions in the context of the restriction of movement during lockdown. It minimised infection risk to non-infected individuals, contributed to the effective use of doctors’ time and assisted in optimising the workload.^3^ The use of WhatsApp has been promoted as a tool to provide clinical assistance and screen potential urgent cases.^4^ Informed by existing literature on the topic, a virtual coordination of care plan was devised to support the patients of PJH in their home-based care setting.

The first COVID-19 case in PJH, diagnosed on 28 June 2020, was a staff member and three days later the first resident tested positive. A WhatsApp group with the nursing sisters and the family physicians was formed to facilitate real-time communication. Additionally, daily meetings were arranged via Zoom. Each of these two communication platforms provided unique features and opportunities. The WhatsApp group facilitated real-time communication where questions, results and clinical images could be shared and responded to. Zoom provided the opportunity to do daily ‘virtual ward rounds’ where patients could be discussed and advice given. Directions on the use of blood tests, oxygen concentrators, antibiotics and steroids were given daily. The information gained by making use of these platforms made it simple for the family physicians to refer patients to hospital if they could not be managed at the PJH.

Between June 2020 and August 2020, only 13 patients out of a total of 160 patients tested positive. The effective and timeous quarantine and isolation procedures contributed to the containment of the disease within PJH. Of the infected patients, two were referred to hospital, four needed oxygen therapy at PJH and only one patient passed away.

## Lessons learnt

**FIGURE 1:**
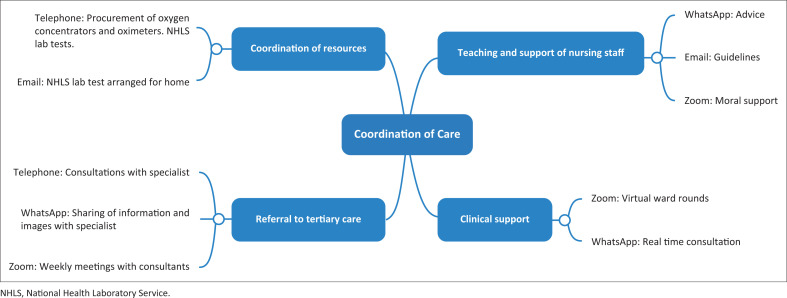
The connections between the roles of the family physician and the technology that made successful virtual care possible.

Through the support of PJH by the Department of Family Medicine, valuable lessons were learnt on being responsive to challenges and thinking outside the box. Without being physically present, it was still possible to fulfil all the key roles of a family physician in the HCA. The use of technology to provide virtual care was a timesaving measure and allowed for real time support that was quicker than the traditional face-to-face interactions. Team building and strengthening of networks in the HCA specifically benefited from the use of virtual platforms and WhatsApp groups. Figure 1 summarises the different roles of the family physician and how technology made them possible.

The nursing manager of PJH summarised the impact of this support during the COVID-19 pandemic well:

‘The fact that only one patient at PJH [*Paul Jungnickel Home*] died from COVID-19 [*coronavirus disease 2019*] is truly remarkable. It would not have been possible without the support of the two family physicians during this crisis time. They empowered us as nurses and assisted in procuring the necessary tools to provide treatment to very special and complex patients in their own safe, home environment. Their moral support was invaluable.’ (S.J.v.V., Nursing manger, 4 May 2021)

## Conclusion

The support to the PJH represents one of the many contributions that family physicians in primary care made during the first wave of the COVID-19 pandemic in Tshwane District. Assistance with planning, co-ordinating clinical care and responsiveness led to an excellent outcome during the COVID-19 outbreak and affirms the importance of the different roles that family physicians play in the South African health context. It also shows the advantages of using innovative strategies and technology to fulfil these different roles.
